# Food web and fisheries in the future Baltic Sea

**DOI:** 10.1007/s13280-019-01229-3

**Published:** 2019-07-26

**Authors:** Barbara Bauer, Bo G. Gustafsson, Kari Hyytiäinen, H. E. Markus Meier, Bärbel Müller-Karulis, Sofia Saraiva, Maciej T. Tomczak

**Affiliations:** 1 Stockholm University Baltic Sea Centre, 106 91, Stockholm, Sweden; 2 Institute of Ecology, Friedrich Schiller University Jena, Jena, Germany; 3 German Centre for Integrative Biodiversity Research (iDiv) Halle-Jena-Leipzig, Deutscher Platz 5e, 04103 Leipzig, Germany; 4 Tvärminne Zoological Station, University of Helsinki, J.A. Palménin tie 260, Hanko, Finland; 5 Department of Economics and Management, University of Helsinki, P.O. Box 27, 00014 Helsinki, Finland; 6 Leibniz Institute for Baltic Sea Research Warnemünde, 18119 Rostock, Germany; 7 Swedish Meteorological and Hydrological Institute, Norrköping, Sweden; 8 Latvian Institute of Aquatic Ecology, Voleru iela 4, 1007 Riga, Latvia; 9 Instituto Superior Técnico, Environment and Energy Section, University of Lisbon, Lisbon, Portugal

**Keywords:** Climate change, Ecopath with Ecosim (EwE), Ecospace, Fisheries, Representative Concentration Pathways (RCP), Shared Socioeconomic Pathways (SSP)

## Abstract

We developed numerical simulations of potential future ecological states of the Baltic Sea ecosystem at the end of century under five scenarios. We used a spatial food web (Ecospace) model, forced by a physical–biogeochemical model. The scenarios are built on consistent storylines that describe plausible developments of climatic and socioeconomic factors in the Baltic Sea region. Modelled species diversity and fish catches are driven by climate- and nutrient load-related changes in habitat quality and by fisheries management strategies. Our results suggest that a scenario including low greenhouse gas concentrations and nutrient pollution and ecologically focused fisheries management results in high biodiversity and catch value. On the other hand, scenarios envisioning increasing societal inequality or economic growth based on fossil fuels, high greenhouse gas emissions and high nutrient loads result in decreased habitat quality and diminished biodiversity. Under the latter scenarios catches are high but they predominantly consist of lower-valued fish.

**Electronic supplementary material:**

The online version of this article (10.1007/s13280-019-01229-3) contains supplementary material, which is available to authorized users.

## Introduction

Environmental scientists are often faced with questions about how changes in anthropogenic drivers of pollution and extractive uses of the marine environments are likely to alter the state of ecosystems and living marine resources. Numerical models, such as global climate models, ecosystem models and species distribution models, offer great potential to address such questions and they are increasingly used in foresight studies and for policy support (Nelson et al. 2009; Jones-Farrand et al. 2011; IPBES 2016). However, a number of uncertainties decrease the predictive capacity of models. Cheung et al. (2016) suggested that one of the major sources of uncertainty in exploring the potential future of living marine resources is scenario uncertainty, defined as uncertainty due to future developments in the natural and anthropogenic drivers of the modelled system. This is important to consider, as climate change, eutrophication and a number of societal developments may interact and significantly alter how human activities affect marine systems (Planque et al. 2010).

Scenario approaches already have a 50-year history of usage in management planning, economic analyses and various scientific disciplines (van Notten et al. 2003) and they are established tools of global climate research. Quantitative descriptions of potential future greenhouse gas emissions [Representative Concentration Pathways, RCPs, Moss et al. (2010)] and global societal developments [Shared Socioeconomic Pathways, SSPs, Hunter and O’Neill (2014)] were initially developed to study climate mitigation and adaptation at global scale. These global futures have occasionally been applied for projecting the future of marine ecosystems (Lam et al. 2016). One of the major pressures on marine ecosystems is exploitation by fisheries. Cheung et al. (2016) suggested that fishing sector-specific storylines need to be developed and combined with climate and broader socioeconomic scenarios to investigate the future states of living marine resources.

**Fig. 1:**
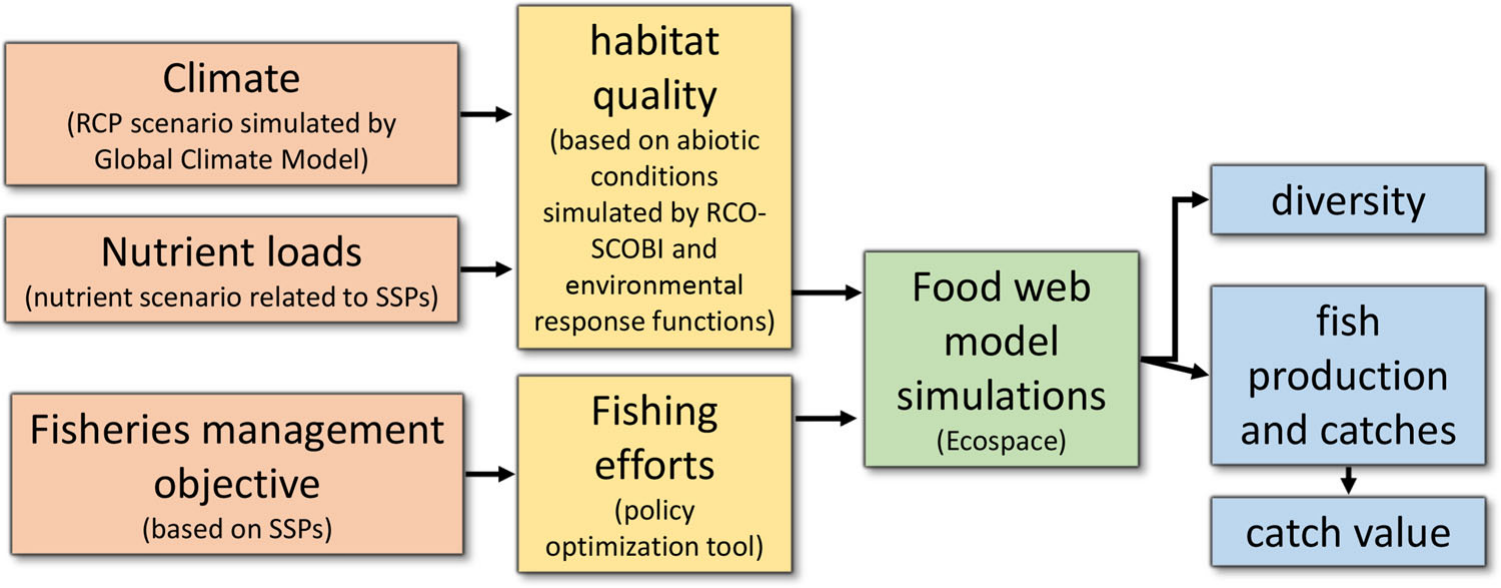
Overview of the modelling framework. The framework consists of scenario assumptions (red boxes), the translation of those assumptions into inputs to the food web model (yellow boxes), food web model simulations (green box) and outputs of those simulations in terms of variables of interest (blue boxes). *RCP* Representative Concentration Pathway, *SSP* Shared Socioeconomic Pathway, *RCO-SCOBI* coupled Rossby Centre Ocean (RCO) model and the Swedish Coastal and Ocean Biogeochemical (SCOBI) model. The calculation of habitat capacity as a basis for habitat quality, fishing effort search using the policy optimization tool and Ecospace simulations are all implemented within the Ecopath with Ecosim software

In this study, we explore potential long-term future states of the Baltic Sea marine food web and fish catches under five integrated scenarios. We use the term ‘integrated’ as each scenario is built on a consistent combination of global climate futures (RCPs) and regional projections of pressures (nutrient loads, fisheries) based on global socioeconomic futures (SSPs). For the latter, we make use of regionally extended socioeconomic narratives developed by Zandersen et al. (2019) for economic sectors that drive nutrient loading and fisheries. Three quantitative nutrient load projections, in combinations with two RCPs, are used as inputs to a coupled physical-biogeochemical model to estimate their impact on the marine environment (Saraiva et al. 2018). The results of that modelling, in combination with fisheries storylines based on Zandersen et al. (2019), are used to drive a spatial food web model to explore the future state of the ecosystem, focusing on biodiversity and fish provision (Fig. 1). We constructed the five integrated scenarios based on five clearly different global futures, in this way aiming to cover plausible limits on some of the key pressures on the environment.

## Materials and methods

### Study system: The central Baltic Sea

The studied area is the central Baltic Sea, a large brackish water body in northern Europe that is subject to many human pressures such as nutrient and other pollution, climate change and fishing (Snoeijs-Leijonmalm et al. 2017). Wide-spread bottom hypoxia, decreasing salinity and increasing temperatures over the last decades significantly affected its biodiversity (Ojaveer et al. 2010; Carstensen et al. 2014; Snickars et al. 2015; Casini et al. 2016). The commercial fisheries in the Baltic Sea are supported by only a few species. Demersal fisheries catch cod (*Gadus morhua*) and flounder (*Platichthys flesus*) using bottom trawls and passive gears such as gillnets. The latter also target central Baltic herring (*Clupea harengus*), which is however caught mostly together with sprat (*Sprattus sprattus*) using pelagic trawls. Pelagic fisheries account for the majority of catches in the region (ICES 2016a).

### Biogeochemical model

Marine food web simulations use outputs from the regional coupled physical–biogeochemical model RCO-SCOBI that comprises the physical Rossby Centre Ocean (RCO) model (Meier et al. 2003) and the Swedish Coastal and Ocean Biogeochemical (SCOBI) model (Eilola et al. 2009). The RCO simulates hydrological processes such as mixing and water transport. In the northern Kattegat open lateral boundary conditions are used, where in case of inflow across the model border temperature, salinity and nutrient values are nudged towards observed climatological profiles. In case of outflow, the boundary conditions are formulated such that anomalies of temperature, salinity and nutrient concentrations may leave the model domain. SCOBI describes the dynamics of nitrate, ammonium, phosphate, oxygen and hydrogen sulphide concentrations (the latter as negative oxygen), three phytoplankton species, zooplankton and detritus (Eilola et al. 2009). RCO-SCOBI is forced by atmospheric surface fields from a regional coupled atmosphere–ocean climate model that is driven by lateral boundary conditions from a General Circulation Model (GCM). Runoff and nutrient loads were generated by a regional hydrological model forced by regionalized atmospheric data from the same GCM. In this study, results based on MPI-ESM-LR (GCM A, https://www.mpimet.mpg.de) are presented. For sensitivity analyses, we also used outputs from the EC-EARTH (GCM B, https://www.knmi.nl). For details, the reader is referred to Saraiva et al. (2018).

### Food web and fisheries model

To model the complete food web and fisheries, we used the Ecopath with Ecosim (EwE) approach (Christensen et al. 2014). EwE models consist of three modules: Ecopath (food web structure, initial conditions and parameters), Ecosim (temporal dynamics) and Ecospace (spatial module). Here we used the EwE model of the central Baltic Sea described by Bauer et al. (2018), but with small modifications (see Supplementary Information S1).

Our EwE model comprises 21 living groups, representing the food web of the offshore Baltic Sea. The groups are phytoplankton, four zooplankton groups, benthic groups, four fish species separated in adults and juveniles, offshore fish-feeding birds and grey seals. It includes ten fishing fleets: active demersal (ACT; such as bottom trawls) in three size categories: < 18 m, 18–24 m, 24–40 m; passive demersal (PAS; such as gillnets, longlines and pots) in three size categories: < 12 m, 12–18 m, 18–40 m; and pelagic (PEL; pelagic trawl and pelagic seine) in four size categories: < 18 m, 18–24 m, 24–40 m, > 40 m.

Ecospace generates species distributions taking into account the underlying food web dynamics, environmental driver maps and the environmental response functions of modelled species and functional groups, as well as fishing impacts (see below). The following environmental driver maps are included in the model, all derived based on the outputs of the RCO-SCOBI model: phytoplankton concentration, summer water temperature 0–10 m, bottom O_2_ saturation, bottom salinity, bottom O_2_ concentration, < 60 m salinity, cod reproductive volume (Table S2).

Ecospace also simulates the relative spatial distribution of fishing efforts by distinct fishing fleets according to a gravity model, meaning that grid cells with higher expected profits are assigned higher efforts (representing here effective fishing days per vessel). The overall magnitude of fishing efforts across the whole area itself is not calculated by Ecospace but needs to be predefined. Here, we set efforts according to fisheries management objectives in each scenario (see section “Fisheries scenarios” below). Efforts are linearly related to fishing mortality rates (*F*). Thus, an effort value of 2 by a given fleet results in a doubling of Fs caused by that fleet on all of its target species compared to the Fs applied in Ecopath (Fig. S2). Total F for a certain species is the sum of the mortality rates caused by each fleet targeting it.

### Greenhouse gas emission and nutrient load scenarios

Combinations of two greenhouse gas emission scenarios and three nutrient load scenarios were simulated by RCO-SCOBI in a previous study (Saraiva et al. 2018). Results from Saraiva et al. (2018) were used to compute environmental driver maps for the Ecospace model in the ‘future’ scenarios, based on average values for the years 2069–2098. For the ‘current’ projections we used average values for the period 2006–2015 under the combinations of RCP4.5 and RCP8.5 scenarios with the two extreme nutrient scenarios WORST and BSAP. In general, changes between RCP scenarios result in changes in all the relevant environmental properties represented in the driver maps, while nutrient load scenarios influence only the oxygen concentrations in the water column and total phytoplankton biomass (see Supplementary Information S2 for examples of driver maps).

Greenhouse gas concentration scenarios used were RCPs 4.5 and 8.5, taken from the latest IPCC assessment report (Moss et al. 2010; IPCC 2014). These scenarios are defined as leading to a radiative forcing of 4.5 Wm^−2^ at stabilization after 2100 and 8.5 Wm^−2^ in 2100. Nutrient load scenarios were the Baltic Sea Action Plan (BSAP), Reference (REF) and a High End nutrient scenario (WORST). The Baltic Sea Action Plan is an international programme between the Baltic Sea countries to improve the ecological status of the Baltic Sea (HELCOM 2007). Thus, in this scenario, nutrient load reduction targets are assumed to be reached by 2020, with loads remaining constant thereafter. In the REF scenario, loads are simulated assuming that the positive and negative impacts of changes in the socioeconomic drivers cancel each other out, but the changing climate still affects air temperature and precipitation. Loads are highest in the WORST scenario, both due to increased precipitation and changes in the socioeconomic drivers of nutrient loading.

### Fisheries scenarios

For each fishery scenario, fleet-specific fishing efforts were calculated, using the ‘Fishing policy search’ tool in EwE. The ‘Fishing policy search’ tool optimizes fishing efforts according to predefined targets. Targets implemented in our study were, using EwE terminology (Table 1): ecosystem structure (‘Nature First’ scenario), net economic value (‘Rich Man, Poor Man’, ‘Growth First’) and employment (‘Isolation’). Optimizing ‘ecosystem structure’ means finding fishing efforts that produce the highest total biomass over all modelled groups, weighted by longevity (i.e. high biomass of groups such as grey seals matter more than those of plankton). This index is related to Odum’s ecosystem maturity index (Odum 1969) and indicates highly efficient biomass transfer to higher trophic levels, i.e. high ecosystem integrity. Optimizing ‘employment’ and ‘net economic value’ correspond to maximizing landed value and landed value minus fleet costs, respectively (Christensen and Walters 2004). Here we used ‘net economic value’ optimization in two ways, either maximized as a systemic objective (‘Growth First’), or for each vessel separately (‘Rich Man, Poor Man’). Economic parameters used in the model are described in Supplementary Information S1. In contrast to the other scenarios, we did not use the ‘Fishing policy search tool’ to find the applicable fishing efforts in our fifth scenario: ‘Constant Compromise’. Here, fishing efforts for all fleets were kept at their current level [average 2006–2013, based on effort data from the European Commission’s Scientific, Technical and Economic Committee (STECF) measured as kilowatt days at sea, for more details see the EwE model description in ICES (2016b)].

**Table 1:** Summary of scenario assumptions

Scenario	RCP	Nutrient scenario	Fisheries management target
Nature First	RCP4.5	BSAP	Maximum ecosystem integrity
Constant Compromise	RCP4.5	REF	None, current efforts used
Isolation	RCP4.5	WORST	Maximum landed value, no vessels > 40 m
Rich Man, Poor Man	RCP8.5	REF	Maximum profit by each vessel
Growth First	RCP8.5	WORST	Maximum profit for total fishery

*RCP* representative concentration pathway. For details see “Materials and methods” section

To avoid the optimization procedure becoming stuck at local maxima, all policy optimizations were started with random fleet efforts and repeated 10 times (Christensen and Walters 2004). As the main patterns of the resulting fishing efforts were similar across the 10 runs (i.e. no bimodal solutions were found), we applied their average in the scenario simulations. All other settings in the ‘Fishing policy search’ tool were left at their default values. During each optimization, we assumed constant environmental (oxygen and temperature) conditions corresponding to the average value of the final 30 years (2069–2098) of the corresponding RCO-SCOBI simulations (for example, the RCP4.5 × BSAP simulation when optimizing for ecosystem integrity, cf. Table 1). The search algorithm was set to run for 15 years with constant efforts, as this corresponds to the average lifetime of a fishing vessel, i.e. this is the strategic planning timescale for fishermen.

### Integrated scenarios

The five integrated scenarios are (Zandersen et al. 2019):

1. Nature First (SSP1)—RCP4.5 climate trajectory, nutrient loads according to BSAP and fisheries optimization to maximize ecosystem integrity. Environmental management focuses on ecological sustainability. International cooperation is strong and the Baltic Sea Action Plan is implemented by 2020. Private–public partnership becomes a prevailing arrangement in the fisheries sector and fisheries become integral instruments of environmental management.
2. Constant Compromise (SSP2)—RCP4.5, Reference nutrient loads and current fishing efforts. This scenario assumes some, but not full, international cooperation on nutrient load management and medium economic growth. Fisheries management continues to be based on the same principles applied today.
3. Isolation (SSP3)—RCP4.5, High End nutrient loads, fisheries maximize employment (landed value), no vessels > 40 m operate. This scenario is characterized by weak global trade and slow economic growth. Nationalism increases and international cooperation on load management fails. Waste water management is not a priority and environmental targets are ignored. Fisheries management targets high catch value, implying both high catch amounts and a larger share of valuable fish, which can be used for human consumption, generating higher local employment. Because of international tensions, there are no large fishing vessels that would operate over large areas and fishing is mostly conducted in territorial waters using small- and middle-sized boats.
4. Rich Man, Poor Man (SSP4)—RCP8.5, Reference nutrient loads, fisheries optimize profit by fleets. This scenario assumes a very unequal society, where most power is in the hands of globally connected elites who run high-tech economies and large-scale, very cost-effective industrial farming. Even though the enforcement of environmental regulations is weak, nutrient loads remain at intermediate level because wealthy societies apply innovative technologies to deal with visible environmental problems and also because the population of the Baltic Sea region declines drastically. Fisheries are unregulated (open access) and each fleet operates to maximize its own profit.
5. Growth First (SSP5)—RCP8.5, High End nutrient loads, fisheries optimize total profit. Conventional economic growth-focused development based on fossil fuels. Nutrient loads increase compared to the previous scenario because of a larger increase in livestock to serve the globally increasing demand for meat and dairy.

Technically, each integrated scenario corresponds to seven environmental driver maps and ten fishing efforts (one per fleet). The maps and efforts approximate the prevailing conditions that would affect the food web around the late twenty-first century under a particular storyline. As a simplification, we ignore future interannual variability and assume these conditions to be static. Our aim was to test the equilibrium responses of the food web and fisheries catches to such conditions. Thus, the model was run for 400 time steps (years) in each scenario to ensure stabilization of the spatial distributions of functional groups and fleets, and presented results are calculated as the average of the last 10 time steps.

### Indicators

We calculate several indicators describing ecosystem state in each scenario. These are habitat quality, fishing pressure, biodiversity, catch amount and relative catch value. Habitat capacity, the variable representing the suitability of an area for a given functional group, is calculated separately for each cell and group based on the environmental drivers in the given cell and the environmental response functions of the given group (Christensen et al. 2014; Bauer et al. 2018). Habitat quality is the average of those values over all groups and spatial cells. Thus, it indicates how suitable the abiotic habitat factors are for the organisms in the food web. We note that food availability is not part of this calculation; thus, potential positive effects of increased algal production under the higher nutrient load scenarios are ignored by this indicator, similarly to negative effects of fishing mortality in cells with high fishing efforts.

Fishing pressure is the average fishing mortality rate (*F*, catches divided by biomass) of all adult fish groups. Biodiversity is here specified as the average number of groups present (biomass > 10 g/km^2^) in each cell. As the model has 21 groups, our biodiversity measure takes a maximal value of 21. The use of a biodiversity index provides added value compared to the habitat quality index as it integrates the effect of biotic interactions on top of environmental suitability in determining the presence of each food web group in a given cell. Relative catch value is calculated as catch amount multiplied by the price of fish. However, as future prices are highly uncertain, we assumed that the relative price per tonne of cod is 1, flounder is 0.35 and herring and sprat is 0.25 (based on their market prices in 2015), and resulting total catch values were scaled so that the highest value was set at 100%. We present the sum of catches and relative catch value of cod and flounder (‘demersal’), and herring and sprat (‘pelagic’). This is done as the exact species composing the fish community may be different at the end of the century from today, but demersal and pelagic species will likely still depend on similar abiotic conditions as they do today (those of the seafloor and the water column, respectively). The two species groups are also likely to remain being fished separately by different fisheries in the future.

### Sensitivity analysis

To test the sensitivity of model results to the GCM applied, we also simulated the integrated scenarios ‘Constant Compromise’ and ‘Rich Man, Poor Man’ based on RCO-SCOBI outputs that were driven by the GCM B. We chose these two scenarios as they are based on RCP4.5 × REF and RCP8.5 × REF, respectively. Thus, we are able to test the effects of using a different GCM assuming both climate change scenarios (RCP4.5 and RCP8.5), combined with an intermediate (REF) nutrient scenario.

To test the sensitivity of the fishing effort optimization to economic data, we repeated optimizations for all four scenarios (all except the Constant Compromise) setting fleet-specific costs to the same value for all fleets, using default settings in EwE (no fixed costs, sailing and other effort related costs are both 40% of revenue).

## Results

### Habitat quality and biodiversity

**Fig. 2:**
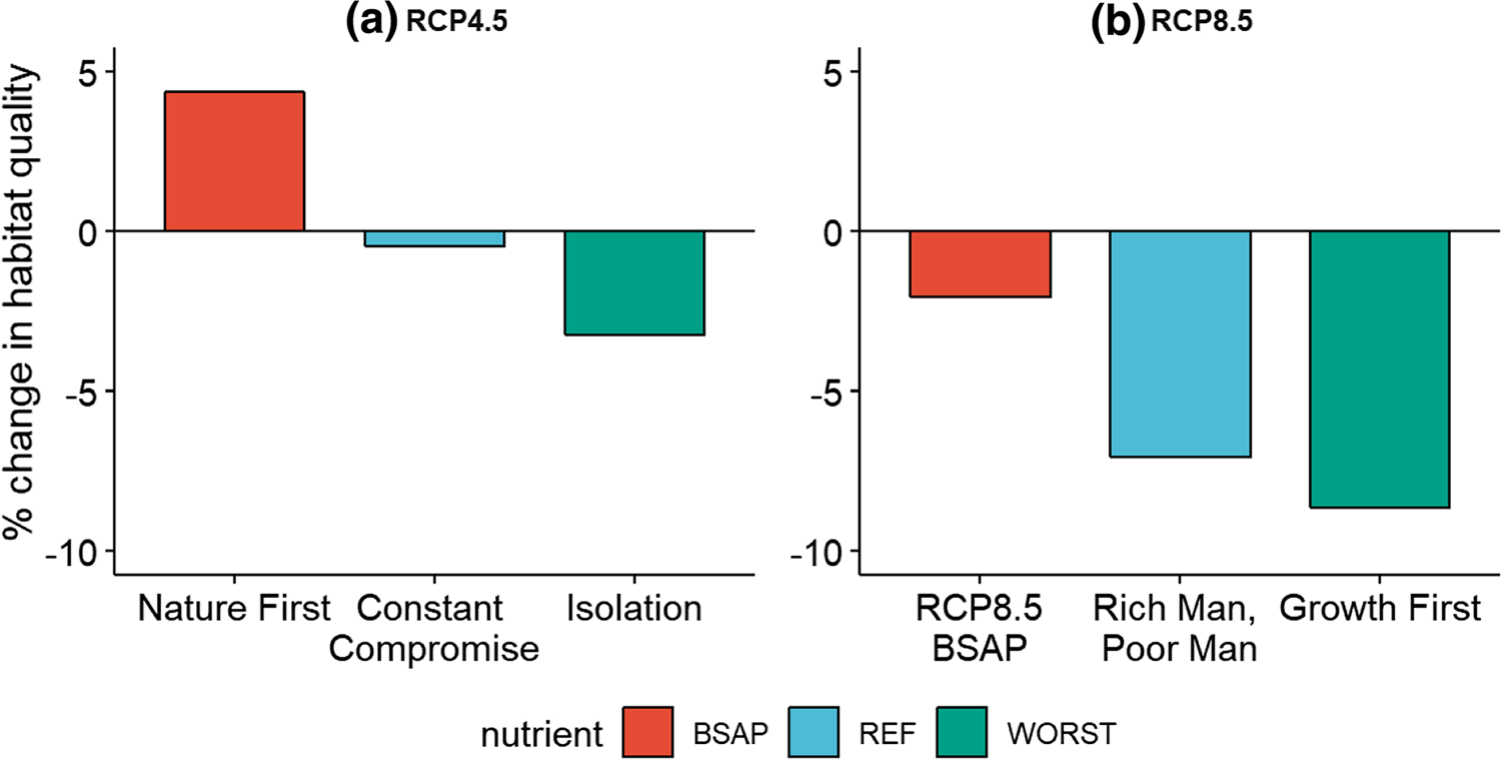
Percentage change in habitat quality in the future scenarios (average 2069–2098), compared to a baseline of simulations corresponding to a ‘current’ (average 2006–2015) state under **a** RCP4.5 and **b** RCP8.5 climate scenarios combined with the nutrient scenarios BSAP (red), REF (blue) and WORST (green). For completeness, we also show habitat quality under a combination of the RCP8.5 climate and the BSAP nutrient scenarios, even though this combination was not simulated in any integrated scenario

Overall habitat quality in the simulated future scenarios is always lower than in simulations corresponding to the ‘current’ state, except in the Nature First scenario (Fig. 2). Under both RCP4.5 and RCP8.5, habitat quality is highest under the BSAP nutrient scenario and lowest under the WORST nutrient scenario, due to negative effects of expanding hypoxia (Fig. S4). Under a given nutrient scenario, habitat quality is always lower under RCP8.5 than under RCP4.5, due to decreasing salinity (Fig. S6). Thus, the decline in habitat quality is especially pronounced under the combination of RCP8.5 and higher nutrient loads.

**Fig. 3:**
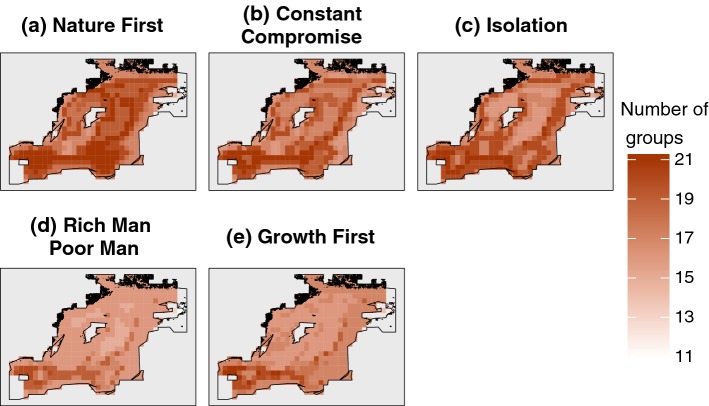
Species richness (measured as number of groups present in each cell) in the integrated scenarios. **a** Nature First, **b** Constant Compromise, **c** Isolation, **d** Rich Man, Poor Man, and **e** Growth First

**Fig. 4:**
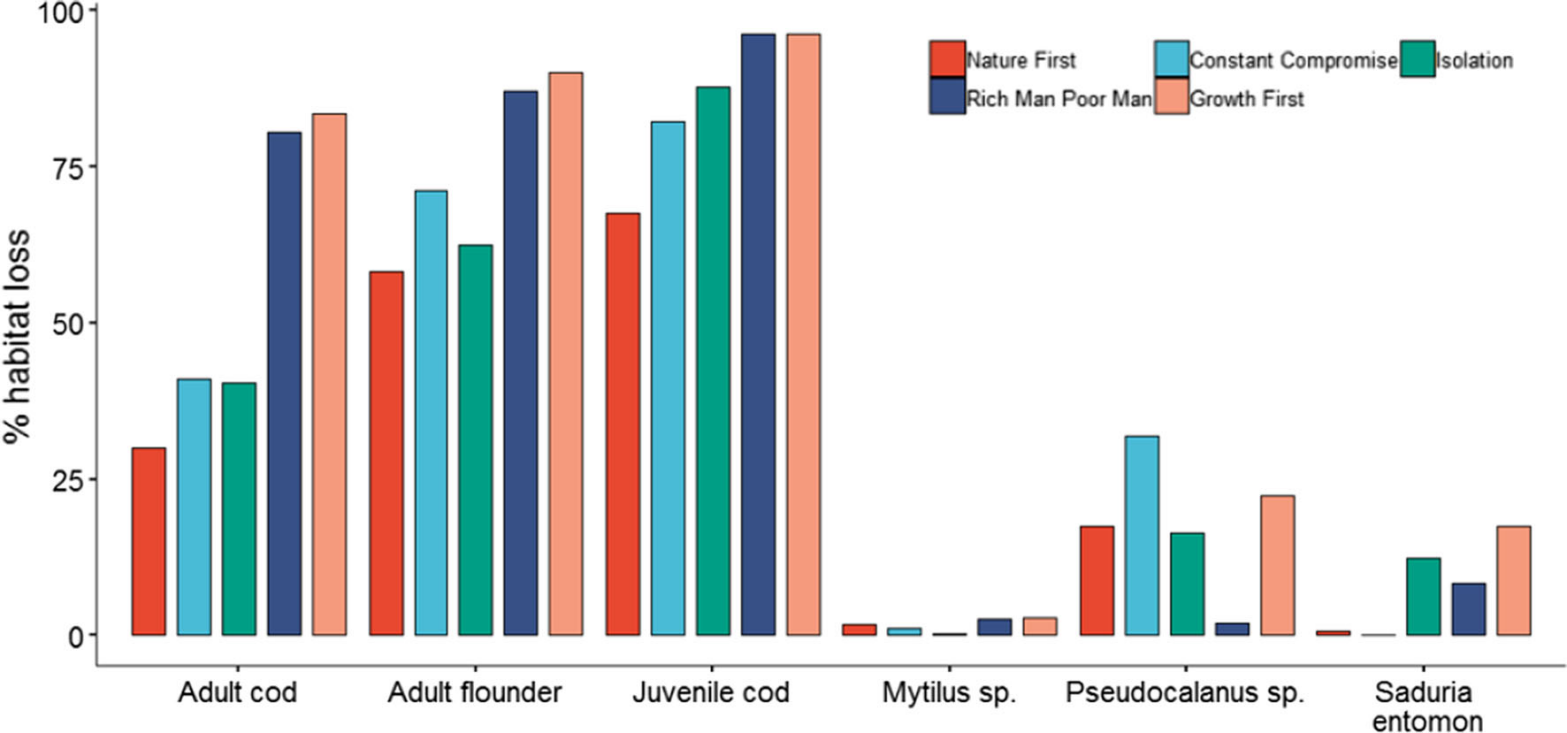
Fraction of habitat lost due to local extinctions by each functional group, defined as percentage of grid cells where that group was absent in each scenario. Groups not shown were present in > 99% of the grid cells in all scenarios. Local extinctions of juvenile flounder were identical to those of adult flounder. Fish-feeding birds became extinct from the whole modelled area in the Rich Man, Poor Man scenario; in other scenarios they were present in every grid cell

Biodiversity, here measured as species richness (Fig. 3), reflects the patterns of habitat quality in the five integrated scenarios (Fig. 2). Biodiversity is highest in the Nature First scenario, especially in the southern Baltic Sea (Fig. 3a). It decreases in the Constant Compromise and Isolation scenarios (Fig. 3b, c), especially in the areas most strongly affected by hypoxia (Fig. S4), largely due to the disappearance of adult and juvenile demersal fish (Fig. 4). Biodiversity decreases further in all areas in the scenarios assuming RCP8.5 (Fig. 3d, e), most strongly around the coast with low salinities (Fig. S6), except for the southwestern Baltic where it remains relatively high. In these scenarios, demersal fish as well as *Mytilus* sp. are lost from further areas (Fig. 4). *Saduria entomon* becomes locally extinct from the largest areas under the scenarios assuming WORST nutrient loads, and less under the scenario assuming Reference nutrient loads and RCP8.5 (Fig. 4). Thus, the distribution of *Saduria* is more restrained by bottom O_2_ conditions than salinity.

Changes in habitat quality alone do not explain all changes in biodiversity. For example, the group *Pseudocalanus* sp. is only sensitive to salinity among the environmental variables (Table S2). Therefore, solely based on its environmental needs, it would be expected to show the same distribution patterns under the same climate scenarios, while being unaffected by nutrient load and fisheries scenarios. Instead, it shows higher local extinction in the scenarios with high pelagic fish biomass (Figs. 4, cf. 5) suggesting that its increased mortality in those scenarios is due to increased predation pressure.

**Fig. 5:**
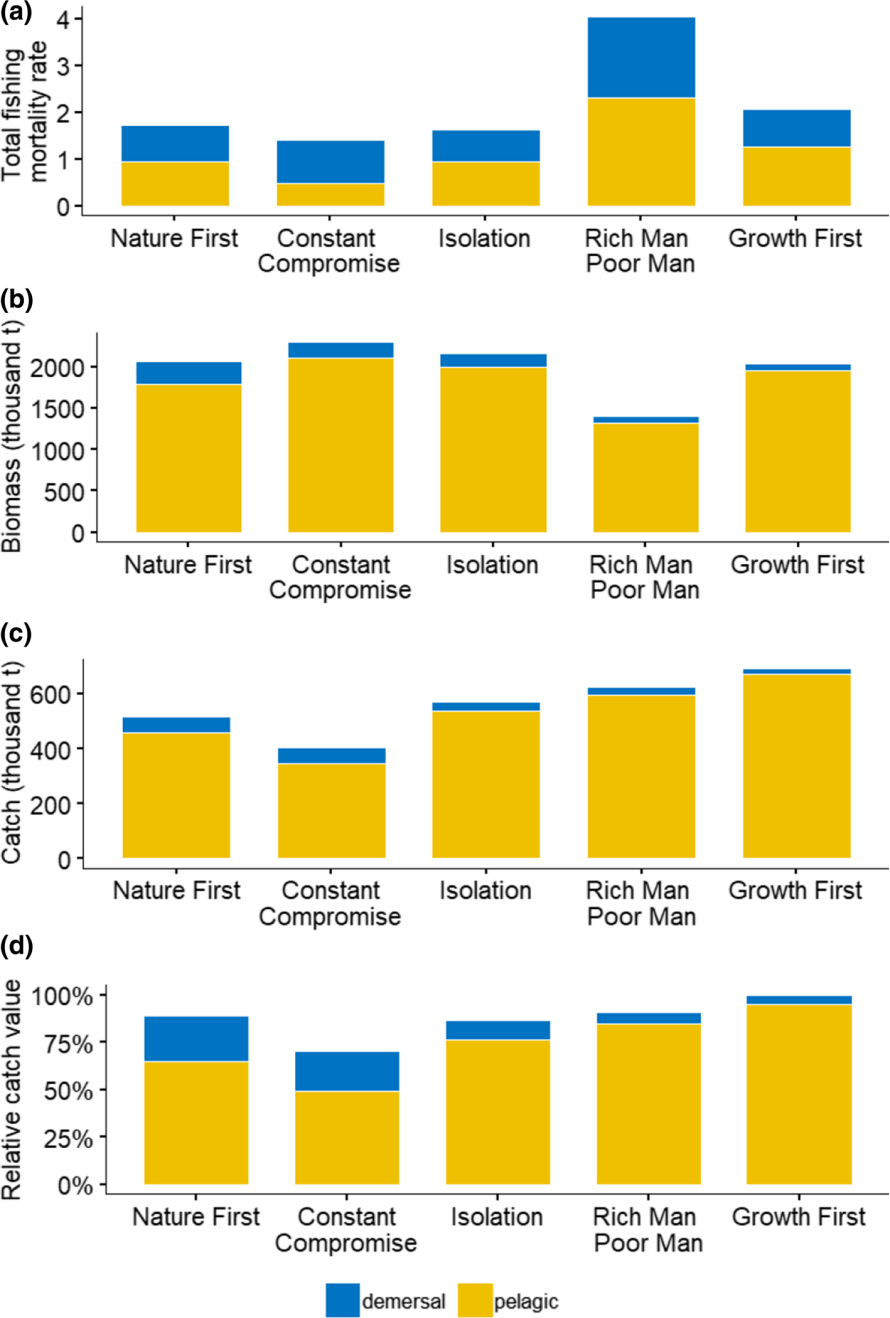
Simulated total **a** fishing mortality, **b** biomass (thousand tonnes), **c** catches (thousand tonnes) and **d** relative catch value in the five integrated scenarios of demersal (blue, cod and flounder) and pelagic (yellow, herring and sprat) groups, including juveniles. For calculation of relative catch value, we multiplied the catches of each species by their relative values, which were 1 for cod, 0.35 for flounder and 0.25 for sprat and herring (based on their relative prices in 2015, Table S1). The scenario with the highest catch value (Growth First) was then assigned 100% relative catch value and catch values of other scenarios were calculated relative to that

### Fisheries

The fishing mortality rate (*F*) caused by both demersal and pelagic fisheries is highest in the ‘Rich Man Poor Man’ scenario, when fisheries are not centrally regulated (Fig. 5a). In all scenarios where fishing efforts were optimized, Fs for pelagic fisheries were higher than for demersal fisheries, in contrast to the Constant Compromise scenario. The latter scenario also had the lowest overall Fs (Fig. 5a).

Fish population biomasses were lowest in the Rich Man, Poor Man scenario (Fig. 5b), even though habitat quality was marginally better than in the Growth First scenario (Fig. 2). In the scenarios assuming RCP4.5, fish biomasses were consistently higher than in those assuming RCP8.5. In the latter, there is very little demersal fish present in the fish community due to decreased habitat quality in most areas (low salinity and bottom O_2_ concentrations, see Supplementary Information S2). In contrast, the ratio of demersal to pelagic fish was highest in the Nature First scenario (Fig. 5b). This is not surprising, as in this scenario fishing efforts are set to increase the ratio of larger sized organisms (‘ecosystem integrity’), including large fish, and habitat quality is also at its highest.

**Fig. 6:**
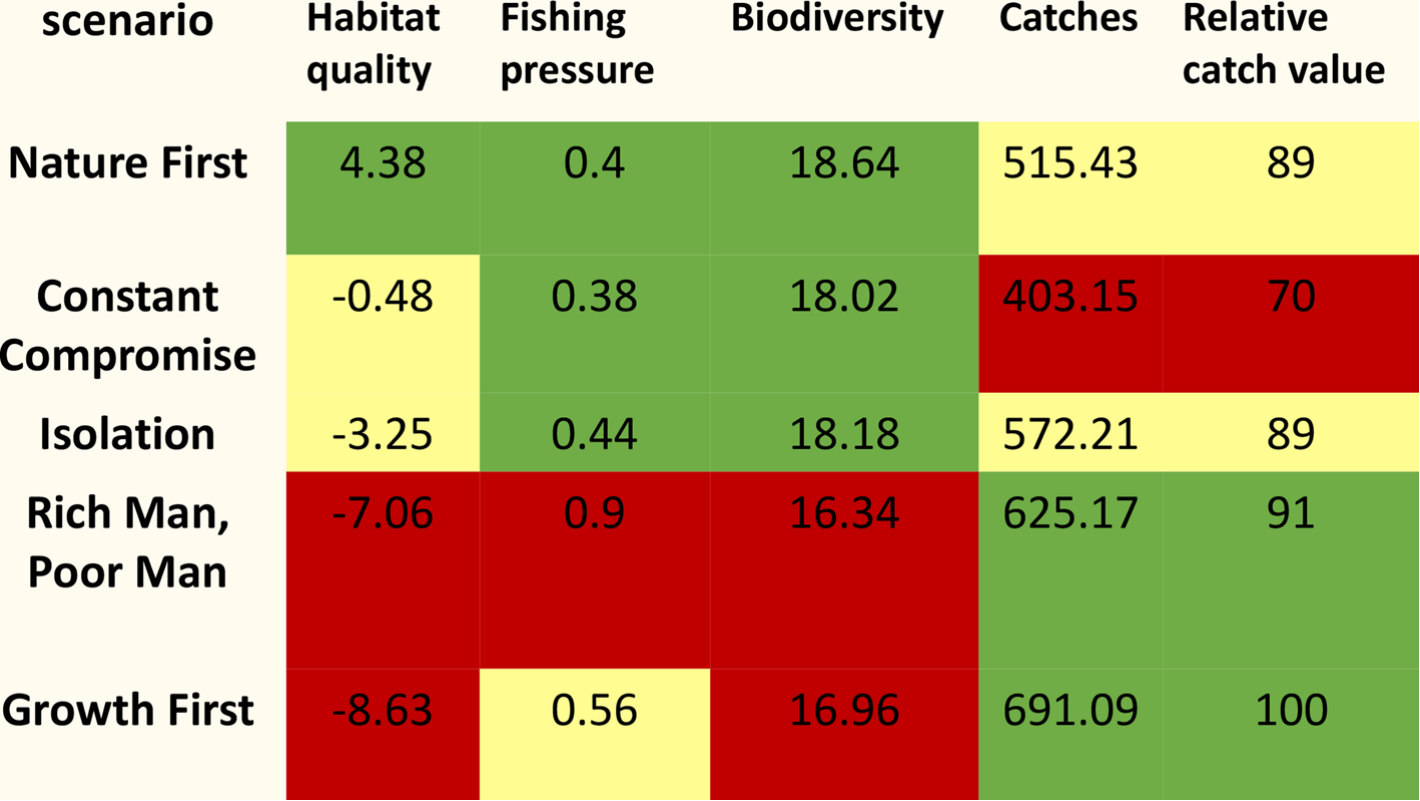
Scenario outcomes in terms of ecological and economic indicators. For visualization purposes, indicator values were assigned the colours red, yellow and green to indicate normatively ‘bad’, ‘intermediate’ and ‘good’ performance, respectively. In the absence of absolute reference points for these indicators, colours are based on relative values from the scenarios categorized on a scale from 0 to 1, where 0 (1) corresponds to the descriptor values in the worst (best) performing scenarios, values < 0.33 were coloured red (‘bad’), 0.33–0.66 yellow (‘intermediate’) and > 0.66 green (‘good’). For fishing pressure, where lower values have a normatively positive meaning, we used inverse colouring. Calculation of indicator values is described in the Materials and Methods section of the main text

Catches (Fig. 5c) and relative catch value (Fig. 5d) depended both on the magnitude of Fs (Fig. 5a) and on fish biomasses (Fig. 5b). Even though Fs in the Rich Man, Poor Man scenario were twice those in Growth First, catches and catch values were slightly lower than in Growth First (cf. Fig. 6). This was due to substantial overfishing in the Rich Man, Poor Man scenario, indicated by low fish biomasses (Fig. 5b). Fs are similar in the Isolation and Nature First scenarios (Fig. 3), resulting in similar catches. These scenarios had the same catch value, which was only marginally lower than in the Rich Man, Poor Man scenario. Catches and catch value were lowest in the Constant Compromise scenario (Figs. 5c, d and 6).

### Sensitivity analysis: Influence of the choice of Global Climate model (GCM) and the economic parameters

In general, scenario simulations based on forcing generated by GCM B showed similar patterns to those based on GCM A. Biodiversity was slightly higher (by 5 and 8%, for Constant Compromise and Rich Man, Poor Man, respectively) in the scenario with GCM B, but smaller than the differences predicted between scenarios (cf. Fig.6, Fig. S8). This increase can probably be explained by higher salinity in simulations based on GCM B over the whole Baltic Proper, especially under RCP8.5, and higher oxygen concentrations in the areas most affected by hypoxic bottoms under RCP4.5 (Fig. S7). Similarly, differences in results due to the choice of GCM were smaller than differences between scenarios with respect to catch amounts and relative catch values (Fig. S9). Simulations based on GCM B mostly resulted in larger demersal fish catches, probably related to the higher salinity (both RCPs) and higher oxygen concentrations (under RCP4.5 only) compared to GCM A-driven simulations.

Fishing effort predictions by the fishing effort optimization tool were sensitive to economic data (Supplementary Information S4). The effort optimization procedure that was used to specify efforts in the integrated scenarios was based on fleet-specific costs and profits. This resulted in very high efforts by large pelagic fleets and small vessels using passive gears in the two profit-based scenarios (Rich Man, Poor Man and Growth First, Fig. S10). In the Nature First scenario, efforts among fleets were equal. In the Isolation scenario, where > 40 m vessels could not operate by definition, high efforts by mid-sized (18–24 m) fleets were found by the optimization routine, in addition to high efforts by vessels using small passive gears. When setting the same costs and profits for all fleets (instead of using fleet-specific costs and profits), the optimization no longer produced consistently high efforts for any type of fleet in any scenario (Fig. S12). The only exceptions were vessels using small passive gears in the Rich Man, Poor Man scenario. This suggests that the original optimization search favoured this fleet not due to its cost parameters but due to the higher price of fish caught by these gears (Table S1). In contrast, among pelagic fleets > 40-m or 18–24-m vessels were originally favoured due to lower costs compared to other fleets (Fig. S3).

## Discussion

This paper presents one of the first applications of long-term, quantitative regional scenarios for a marine ecosystem based on global scenarios. It also considers both social and ecological aspects which are often not integrated (Österblom et al. 2013). We estimated the magnitude of potential changes in important factors affecting ecosystem state (habitat quality, fishing), the response of the marine food web (biodiversity) and economic benefits derived from the sea (catches and catch value) under five different scenarios for the Baltic Sea environment and its management (Fig. 6). Our normatively positive scenario (Nature First) involves ecological sustainability and social connectivity. In contrast, the other, more dystopian, scenarios entail increased pressures on the system due to increasingly effective exploitation aided by technologies (Growth First and Rich Man, Poor Man), or through lack of ability or will to improve the state of the environment because of a lack of social cohesion (Isolation and Rich Man, Poor Man).

Our model suggests that future evolution of biodiversity is heavily dependent on both global factors (climate) and regional socioeconomic developments (driving nutrient pollution and fishing effort), in agreement with Niiranen et al. (2013). Regional management actions to reduce nutrient loads could significantly improve bottom oxygen conditions (Meier et al. 2012a, b; Neumann et al. 2012), which is one of the key factors required to ensure suitable habitat conditions for many Baltic Sea organisms (Snoeijs-Leijonmalm et al. 2017). However, salinity, affected by global climate, also has a significant impact on species distributions (Mackenzie et al. 2007; Gogina and Zettler 2010; Eero et al. 2015). Consequently, high global greenhouse gas emissions may compromise local efforts to safeguard biodiversity.

It should be noted that the model does not represent the full range of species diversity found in the offshore Baltic Sea. Thus, our conclusions regarding future changes in diversity are somewhat limited. In addition, our approach only shows how the current ecosystem would respond to constant future conditions at the end of century. However, this response may differ from that produced by gradually changing conditions. In addition, we ignore individual-level responses to climate change and introduction of novel species, both of which could have potentially large impacts on ecosystem processes in the future (Almqvist and Strandmark 2010; Pörtner and Peck 2010).

Our approach also ignores some important processes shaping species assemblages related to natural variability, such as disturbances and stochasticity. The effects of spatio-temporal variability in abiotic environmental conditions on the food web and fisheries can be studied using the spatio-temporal framework in EwE (Christensen et al. 2014). This framework was applied using an earlier version of our model by Bauer et al. (2018), who studied the effects of three nutrient load scenarios on the ecosystem, assuming no changes in fishing efforts compared to today. The integrated scenarios described by Zandersen et al. (2019) used in this study provide descriptions of regional drivers, such as fisheries management, over the long term. However, they do not describe the temporal development of the drivers from their current state to their state in the scenarios explicitly, which precludes the use of a spatio-temporal framework here. Because of dynamic feedbacks between environmental conditions, food web processes, fishermen behaviour and environmental policy, it is challenging to develop spatially and temporally explicit quantitative projections of the food web and fisheries under future scenarios. This challenge can potentially be addressed by end-to-end or whole-of-ecosystem models (Fulton et al. 2014; Bossier et al. 2018), equation-free modelling (Deangelis and Yurek 2015) and by synthesizing information from several, individually limited scenario studies such as ours in combination with knowledge of the drivers of changes in the past (Uusitalo et al. 2016).

The EwE model only includes one phytoplankton group, and is therefore unable to simulate species shifts within the phytoplankton community under the future scenarios. In the Baltic Sea, those shifts are expected to have negative consequences for consumers under increasing eutrophication (Lehtiniemi et al. 2002; Neumann et al. 2012; Suikkanen et al. 2013). For our study, this implies that fish catches may be overestimated under the high, and possibly even the intermediate, nutrient load scenarios. Thus, improving the representation of lower trophic levels in the context of whole food web modelling would be an important area for further developments.

The Rich Man, Poor Man scenario reproduces the ‘tragedy of the commons’ situation (Hardin 1968), where fisheries are not regulated and the fish stock is overutilized by profit-maximizing fishermen acting in their own self-interest. In particular, under this scenario demersal fish reach very low population levels that would make them vulnerable to stochastic extinctions (Lande et al. 2003; Myers and Worm 2005). The optimization procedure often resulted in excessive fishing rates, as already shown by Christensen and Walters (2004). Our model results suggest that even a degraded ecosystem may be able to support high levels of fish catches (Fig. 6). However, in the Baltic Sea these catches are likely to consist mostly of pelagic fish, which are more resistant to changes in oxygen conditions, but less valued today than demersal fish such as cod and flounder. Results from the Nature First scenario show that under a combination of favourable abiotic conditions (increased bottom oxygen concentrations and higher salinity) the ecosystem would be able to support catches of high value even when fishing efforts remain at relatively low levels.

As a further step, our projected catches could be combined with an analysis of human population development and dietary changes around the Baltic Sea, as well as future levels of pollutants in fish, to estimate the societal value of fisheries from the perspective of food provision in each scenario. However, we should note that our optimal fishing effort estimations are very sensitive to economic parameters. For example, the fleets selected by optimization depended on relative fish prices. Small differences in economic parameters (e.g. between large pelagic and other pelagic trawls) resulted in substantial changes in predicted efforts. Thus, more work is necessary to understand and quantitatively model the processes driving those economic parameters.

We acknowledge that this study represents only a limited sample from plausible combinations of possible future developments of climate, nutrients and fisheries management, and ignores some other pressures on the Baltic Sea environment. A future expansion of the scenarios, either qualitatively or quantitatively, could consider additional environmental, social and economic trends and include plausible projections of technological development, which could substantially change the economic conditions of fisheries. The analysis of the likelihood of extreme events under each scenario as provided by Pinnegar et al. (2006) was out of scope for this study. Also, we did not anticipate huge changes that would completely alter people’s relationship to the environment, which are better addressed using qualitative scenario studies, such as the one by Merrie et al. (2018).

The modelled scenarios could be expanded in the future to incorporate key uncertainties that are not accounted for here. For example, organic pollutants are not represented, but in principle could be handled within the EwE simulation framework (Walters and Christensen 2018). Other key sectors where qualitative trends were described by Zandersen et al. (2019) include aquaculture, electricity production and marine transport.

## Conclusion

The uncertainty of long-term model projections is huge, but scenario studies can assist consideration of possible futures, so that they can be planned for in structured ways. We do not consider our scenario results to be predictions of the future. Instead, we see their value in highlighting the differences among various future development pathways, based on those environmental pressures best described by scientific knowledge (climate and nutrient load effects, fisheries). We hope that the scenarios, besides providing the basis for future, more detailed studies, may prove useful for development of long-term environmental management strategies (IPBES 2016).

## Electronic supplementary material

Below is the link to the electronic supplementary material.

Supplementary material 1 (PDF 548 kb)
