# Supplementary material for: Food web and fisheries in the future Baltic Sea
Source: Ambio. 2019 Jul 26;48(11):1337–49. doi: 10.1007/s13280-019-01229-3 (PMC6814650; doi:10.1007/s13280-019-01229-3)
Supplement: Supplementary file 1 — Supplementary material 1 (PDF 548 kb) [file 13280_2019_1229_MOESM1_ESM.pdf]

Ambio

Electronic Supplementary Material

Title: **Food web and fisheries in the future Baltic Sea**

S1 Ecospace model parameterization

This section describes all modifications of parameters compared to the model version used in Bauer et al. (2018). We decreased the adult cod production/biomass parameter according to Svedäng and Hornborg (2017) and introduced negative biomass accumulation to maintain mass-balance in the model. We have modified the juvenile cod diet composition: the ratio of juvenile sprat was decreased and those of *Saduria entomon* and mysids were increased. These prey are typically eaten by cod too small to be sampled in the stomach sampling program and therefore they are probably underrepresented in the stomach database that we used to parameterize cod diet (ICES 2016). The ratio of sprat in the diet of adult cod was set according to stomach content data, not adjusted according to MSVPA results on sprat mortality as has been done in ICES (2016). Finally, the extent of hypoxic areas was used as time series forcing on *Saduria entomon*, other macrobenthos (Sarvala 1971) and cod feeding (Eero et al. 2015; Köster et al. 2017) as opposed to mysids only. More detail of these changes are found in Bauer et al. (*submitted*).

As the RCO-SCOBI model did not provide hindcast values for environmental parameters for the period 2006-2016, while a suitable fitting period for the EwE model would be 2004-2016, settings for the vulnerability multiplier parameter (*v*) were not determined by formal fitting. Instead, we compared previously fitted versions of the model to various environmental forcing data, and found that they only differed in the type of control (defined based on the v value as 1-<2: bottom-up, 2-10: mixed, 10<: top-down) for four out of seventy-seven modelled predator-prey pairs (juvenile herring- other zooplankton, other zooplankton - phytoplankton, *Acartia sp.* -phytoplankton, *Pseudocalanus sp.* - phytoplankton). For these pairs we assumed the default vulnerability value of 2 (mixed control).

We updated fleet selectivities and cost parameters based on the most recent available economic data, so that our future fishing policy search results (see the *'Fisheries scenarios'* section in the main manuscript) are based on the most current information. Fleet catches (Fig.

S1, Table S1) were parametrized based on landings and discards data from 2016 (https://datacollection.jrc.ec.europa.eu/, accessed 05-10-2017); fish prices (Table S2) and fleet costs (Figure S3) are based on data from 2015 (‘2017-07_STECF 17-12 - EU Fleet Economic and Transversal data’, downloaded 13-11-2017), using the same approach as in (Bauer et al. 2018).

Ecospace settings were as described in Bauer et al. (2018). The exceptions are the environmental response functions of cod, for which the salinity response was set as in Borgstrøm et al. (2007) and the oxygen response as in Casini et al. 2016 (Table S2). We set effort multipliers to the default for all fleets because the value of 5 for pelagics, as applied in Bauer et al. (2018), caused dynamic instability in sprat and herring.

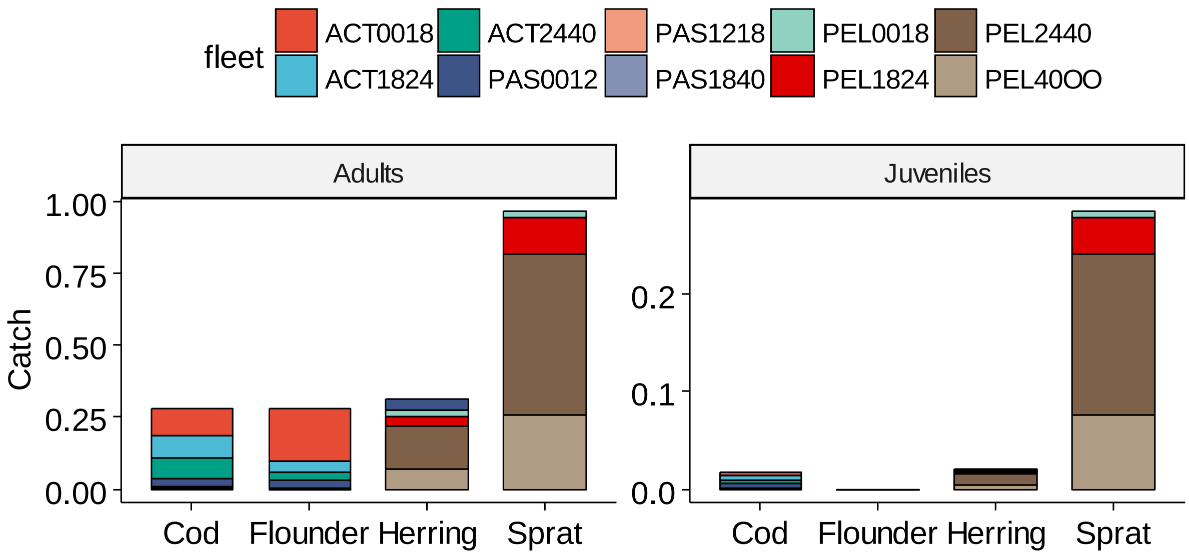

Fig. S1. Catches (landings and discards) for each species by each fleet. While the total amount of catches corresponds to values in 2004 (to correctly represent biomass flows to the fishery in the Ecopath model that is set up to represent 2004 flows), the distribution of catches among fleets, determining fleet selectivity in the future simulations, was set based on data from 2016.

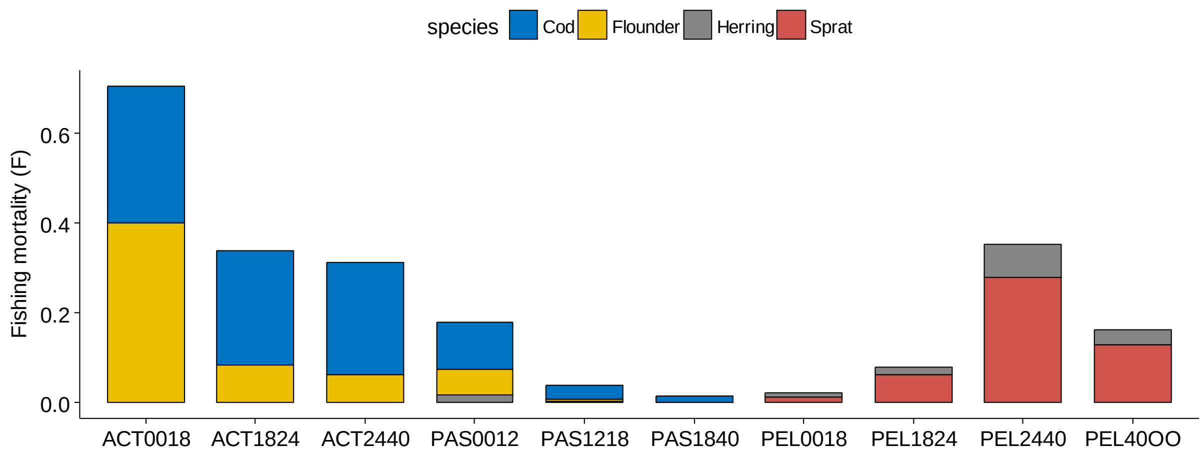

Figure S2 Fishing mortality caused by each fleet in the Ecopath model. Calculated in the Ecopath model as catches (Fig S1) divided by biomass. Fishing efforts applied in the simulations are implemented as multipliers on fishing mortalities shown on this figure. Thus, fishing mortalities applied in the scenario simulations (shown summed up across species for each fleet on S11 and summed up across fleets and species for demersal and pelagic fish groupson Fig. 4) are the product of fishing mortalities shown on this figure and fleet-and scenario- specific fishing efforts (see Section S4 on how fishing efforts were defined in each scenario).

Table S1 Fish prices (EUR/tonne). These are used to calculate revenues by the 'Fishing policy optimization' tool. The abbreviations under 'fleet' comprise letters representing the type of fleet (ACT: active demersal, PAS: passive demersal, PEL: pelagic) and numbers representing the upper and lower limits of vessels size in meters, where 40OO means > 40 m.

Fleet	Juvenile cod	Adult cod	Herring	Sprat	Flounder
ACT0018	640.8525	961.3749			353.1746
ACT1824	694.3859	1041.683			380.4499
ACT2440	662.7642	994.2457			413.5484
PAS0012	791.5497	1187.443	269.6288		389.1725
PAS1218	740.2148	1110.433	370.2484		398.6196
PAS1840	714.4694	1071.811			286.6233
PEL0018			281.8091	275.0491	
PEL1824			269.7256	227.2936	
PEL2440			289.9081	271.9851	
PEL40OO			289.9123	265.026	

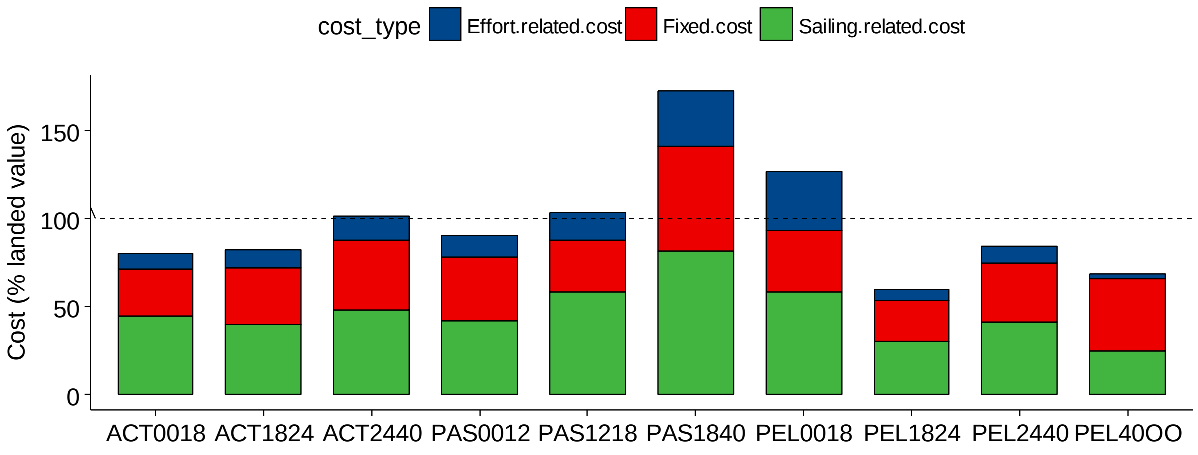

Figure S3 Fleet segment-specific costs. The profit (expressed as % landed value) is the difference between total costs and landed value, i.e. the distance between the top of the barplots and the dashed line. Bars crossing the dashed line indicate non-profitable fleets.

Table S2 Ecospace group parameters: modelled groups, environmental drivers of their distributions, parameters and shape (LS: left-shoulder, T: trapezoid, RS: right-shoulder, see Fig. S8) of environmental response functions and literature sources. The choice of shape shows if the environmental driver in the Baltic Sea has been described to encompass the entirety of the groups' preferred range and values above and below that (trapezoid shape) or the group is only possibly limited by that driver because of too high (left-shoulder) or too low (right-shoulder) values in that ecosystem. Parameters of response functions describe the following ecologically relevant values of environmental drivers: absolute minimum below which the group cannot exist (Min_{Abs}), lower and upper limit of the optimal range (Min_{Opt} and Max_{Opt}, respectively), maximum value above which the group cannot exist at the given location (Max_{Opt}).

**Group name**	**Environmental driver (unit)**	**ERF shape**	**Min_{Abs}**	**Min_{Opt}**	**Max_{Opt}**	**Max_{Abs}**	**reference**
Adult and juvenile cod	Below 60 m salinity	RS	7/9	11/13	-	-	Borgstrøm et al. (2007)
	Bottom O2 concentration (ml/l)	RS	40	65	-	-	Casini et al. (2016)
Juvenile cod	Cod reproductive volume ($m^3 * 10^{-9}$)	RS	0	0.3	-	-	Values set assuming that the optimum codRV range starts at values similar to those seen in SD25

							(which is the only area currently suitable for cod reproduction, Eero et al., 2012)
Juvenile herring, Adult herring, Adult sprat	No environmental response function.						
Juvenile sprat	Average summer (June-October) temperature in upper 10 m water layer (˚C)	RS	0	20	-	-	Baumann et al. (2006)
Juvenile and adult flounder	Bottom O2 saturation (%)	RS	20	40	-	-	Tallqvist et al. (1999)
	Bottom salinity	RS	8	9	-	-	Nissling et al. (2002) cit. in Jokinen et al. (2015)
Saduria entomon	Depth (m)	T	0	24	52	300	Gogina and Zettler (2010)
	Bottom salinity	T	1	7	10	25	
	Bottom O_2 saturation (%)	RS	8	48	-	-	Johansson (1997; Johansson (1999)
Mytilus sp.	Depth (m)	T	0	8.5	21	99	Gogina and Zettler (2010)
	Bottom salinity	T	5	10	21	30	
	Bottom O_2 concentration (ml/L)	RS	2	2	-	-	Karlson et al. (2002)
Macoma b.	Depth (m)	T	0	11	26	140	Gogina and Zettler (2010)
	Bottom salinity	T	2	9	20	33	
	Bottom O_2 concentration (ml/L)	RS	0	2	-	-	Karlson et al. (2002)
Other macrobenthos	Depth (m)	LS	0	0	50	300	estimate based on Gogina and Zettler (2010)
	Bottom O_2 concentration (ml/L)	RS	0	2	-	-	Karlson et al. (2002)
Meiobenthos, Other zooplankton, phytoplankton, Detritus	No environmental response function.						
Mysids	Bottom O_2 concentration (ml/L)	RS	0	2	-	-	Karlson et al. (2002)

Pseudocalanus sp.	Below 60 m salinity	RS	8	9.5	-	-	Margonski & Calkiewicz, (*in prep.*)

References

Bauer, B., H. E. M. Meier, M. Casini, A. Hoff, P. Margoński, A. Orio, S. Saraiva, J. Steenbeek, et al. 2018. Reducing eutrophication increases spatial extent of communities supporting commercial fisheries: a model case study. *ICES Journal of Marine Science*. doi:10.1093/icesjms/fsy003.

Baumann, H., H. H. Hinrichsen, R. Voss, D. Stepputtis, W. Grygiel, L. W. Clausen, and A. Temming. 2006. Linking growth to environmental histories in central Baltic young-of-the-year sprat, Sprattus sprattus: An approach based on otolith microstructure analysis and hydrodynamic modelling. *Fisheries Oceanography* 15: 465–476. doi:10.1111/j.1365-2419.2005.00395.x.

Borgstrøm, R., A. Espersen, K. Geitner, H. H. Hinrichsen, K. Hüssy, G. Kraus, C. Kvaavik, F. Köster, et al. 2007. *Pelagic habitat mapping: A tool for area-based fisheries management in the Baltic Sea*. BALANCE.

Casini, M., F. Käll, M. Hansson, M. Plikshs, T. Baranova, O. Karlsson, K. Lundström, S. Neuenfeldt, et al. 2016. Hypoxic areas, density-dependence and food limitation drive the body condition of a heavily exploited marine fish predator. *Royal Society Open Science* 3: 160416. doi:10.1098/rsos.160416.

Eero, M., M. Vinther, H. Haslob, B. Huwer, M. Casini, M. Storr-Paulsen, and F. W. Köster. 2012. Spatial management of marine resources can enhance the recovery of predators and avoid local depletion of forage fish. *Conservation Letters* 5: 486–492. doi:10.1111/j.1755-263X.2012.00266.x.

Eero, M., J. Hjelm, J. Behrens, K. Buchmann, M. Cardinale, M. Casini, P. Gasyukov, N. Holmgren, et al. 2015. Eastern Baltic cod in distress: Biological changes and challenges for stock assessment. *ICES Journal of Marine Science* 72: 2180–2186. doi:10.1093/icesjms/fsv109.

Gogina, M., and M. L. Zettler. 2010. Diversity and distribution of benthic macrofauna in the Baltic Sea. Data inventory and its use for species distribution modelling and prediction. *Journal of Sea Research* 64: 313–321. doi:10.1016/j.seares.2010.04.005.

ICES. 2016. Report of the Baltic Fisheries Assessment Working Group (WGBFAS), 12- 19 April 2016, ICES HQ, Copenhagen, Denmark. ICES CM 2016/ACOM:11: 593.

Johansson, B. 1997. Behavioural response to gradually declining oxygen concentration by Baltic Sea macrobenthic crustaceans. *Marine Biology* 129: 71–78. doi:10.1007/s002270050147.

Johansson, B. 1999. Influence of oxygen levels on the predatory behaviour of the isopod Saduria entomon. *Marine And Freshwater Behaviour And Physiology* 32: 223–238. doi:10.1080/10236249909379051.

Jokinen, H., H. Wennhage, A. Lappalainen, K. Ådjers, M. Rask, and A. Norkko. 2015. Decline of flounder (Platichthys flesus (L.)) at the margin of the species' distribution range. *Journal of Sea Research* 105: 1–9. doi:10.1016/j.seares.2015.08.001.

Karlson, K., R. Rosenberg, and E. Bonsdorff. 2002. Temporal and Spatial Large-Scale Effects of Eutrophication and Oxygen Deficiency on Benthic Fauna in Scandinavian and Baltic Waters -a Review. *Oceanography and Marine Biology: an Annual Review* 40: 427–489. doi:10.1201/9780203180594.ch8.

Köster, F. W., B. Huwer, H. H. Hinrichsen, V. Neumann, A. Makarchouk, M. Eero, B. V. Dewitz, K. Hüssy, et al. 2017. Eastern Baltic cod recruitment revisited - Dynamics and impacting factors. *ICES Journal of Marine Science* 74: 3–19. doi:10.1093/icesjms/fsw172.

Nissling, A., L. Westin, and O. Hjerne. 2002. Reproductive success in relation to salinity for three flatfish species, dab (Limanda limanda), plaice (Pleuronectes platessa), and flounder (Pleuronectes flesus), in the brackish water Baltic Sea. *ICES Journal of Marine Science* 59: 93–108. doi:10.1006/jmsc.2001.1134.

Sarvala, J. 1971. Ecology of Harmothoe sarsi (Malmgren) (Polychaeta, Polynoidae) in the northern Baltic area. *Ann. Zool. Fenn.* 8: 231–309.

Svedäng, H., and S. Hornborg. 2017. Historic changes in length distributions of three Baltic cod (Gadus morhua)

stocks: Evidence of growth retardation. *Ecology and Evolution* 7: 6089–6102. doi:10.1002/ece3.3173.

Tallqvist, M., E. Sandberg-Kilpi, and E. Bonsdorff. 1999. Juvenile flounder, Platichthys flesus (L.), under hypoxia: Effects on tolerance, ventilation rate and predation efficiency. *Journal of Experimental Marine Biology and Ecology* 242: 75–93. doi:10.1016/S0022-0981(99)00096-9.

S2 Selected environmental driver maps under current conditions and in future scenarios

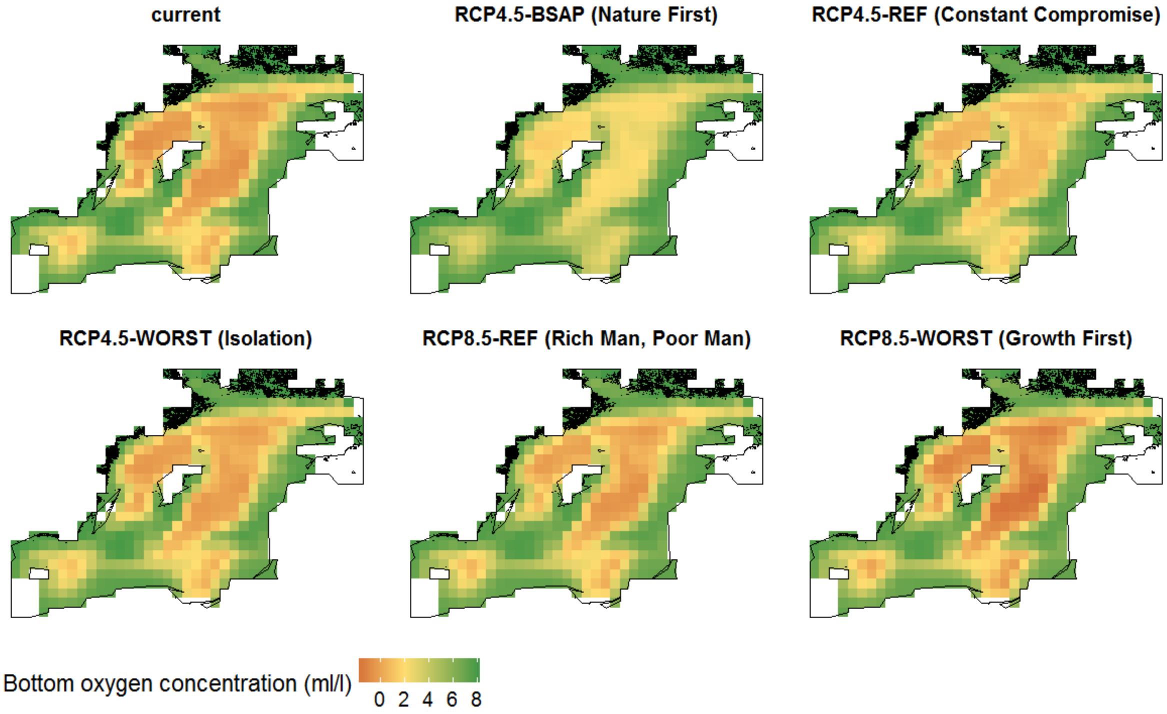

Figure S4 Maps of bottom oxygen concentration, in the 'current run' (average values 2006-2015) and in the 5 scenarios where we used combinations of RCP4.5/RCP8.5 and the Baltic Sea Action Plan (BSAP), Reference (REF) and WORST nutrient scenarios. Maps were generated by aggregating outputs from the RCO-SCOBI model to the spatial resolution and extent of the Ecospace model, from average maps from the final 30 years (2069-2098) simulated by RCO-SCOBI. Negative oxygen concentrations indicate the presence of H_2S. Oxygen <2ml/l is considered hypoxic.

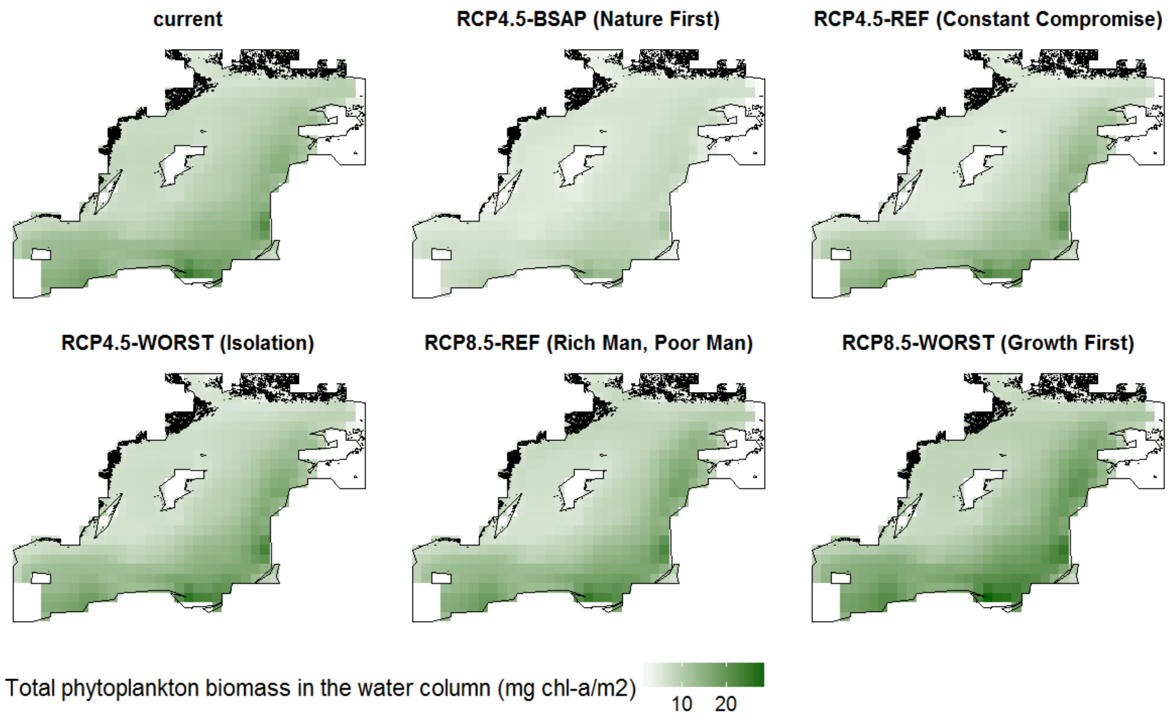

Figure S5 Maps of total phytoplankton concentration (mg chla/m2), in the 'current run' (average values 2006-2015) and in the 5 scenarios where we used combinations of RCP4.5/RCP8.5 and the Baltic Sea Action Plan (BSAP), Reference (REF) and WORST nutrient scenarios. Maps were generated by aggregating outputs from the RCO-SCOBI model to the spatial resolution and extent of the Ecospace model, from average maps from the final 30 years (2069-2098) simulated by RCO-SCOBI.

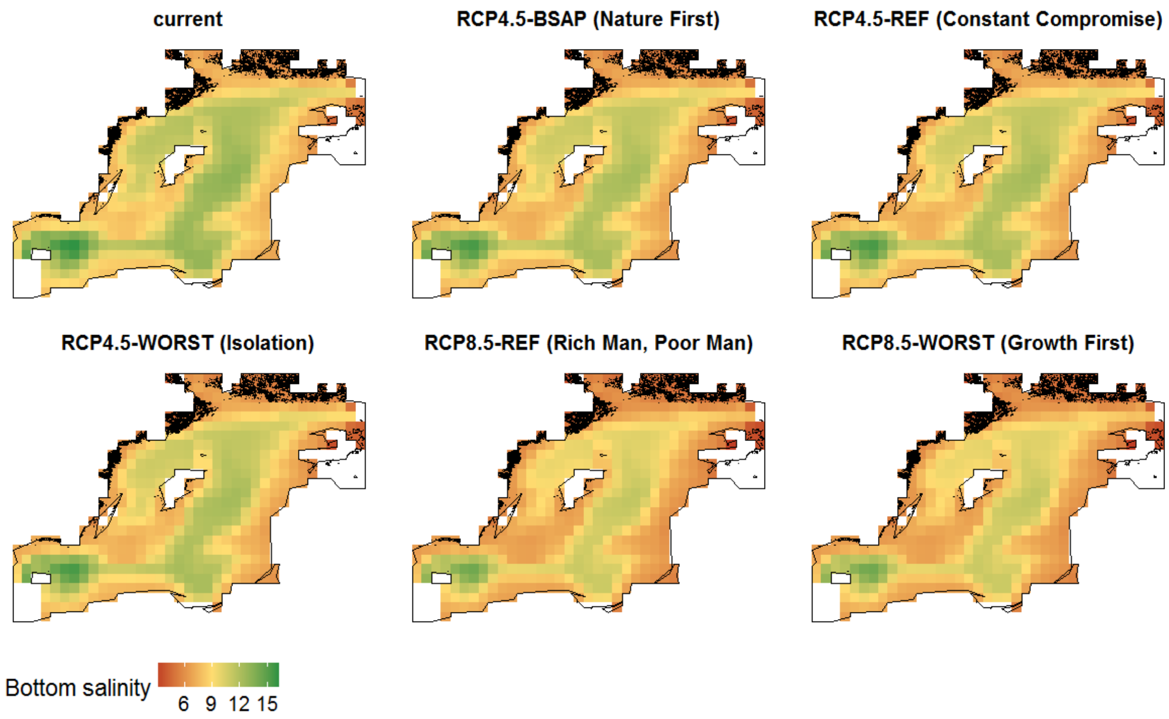

Figure S6 Maps of bottom salinity (psu), in the 'current run' (average values 2006-2015) and in the 5 scenarios where we used combinations of RCP4.5/RCP8.5 and the Baltic Sea Action Plan (BSAP), Reference (REF) and WORST nutrient scenarios. Maps were generated by aggregating outputs from the RCO-SCOBI model to the spatial resolution and extent of the Ecospace model, from average maps from the final 30 years (2069-2098) simulated by RCO-SCOBI.

S3 Supplementary figures - Sensitivity to GCM used

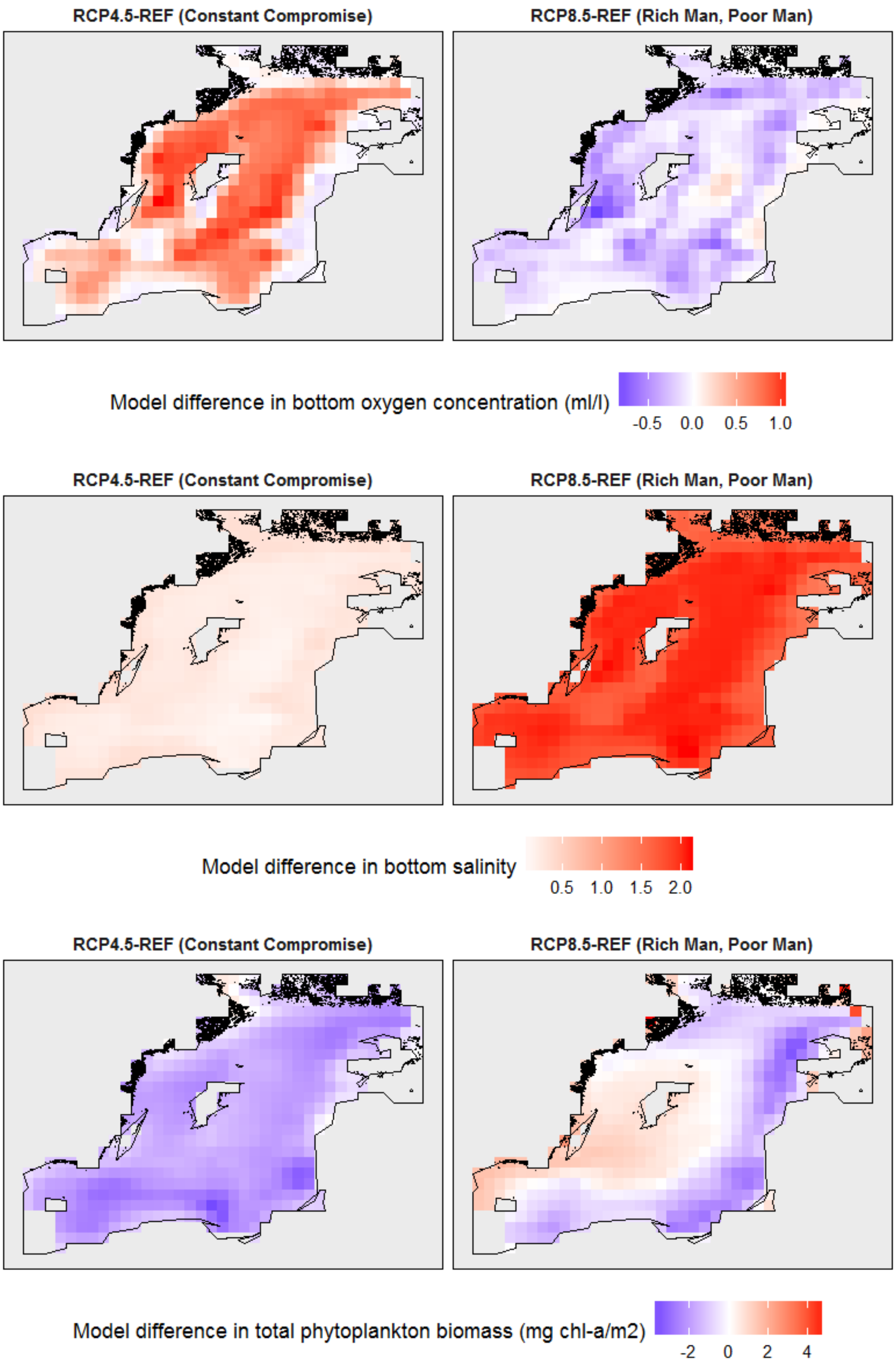

Fig. S7 Differences in environmental drivers calculated as GCM B-GCM A predicted values (Fig. S4-6) in two scenarios: Constant Compromise and Rich Man, Poor Man.

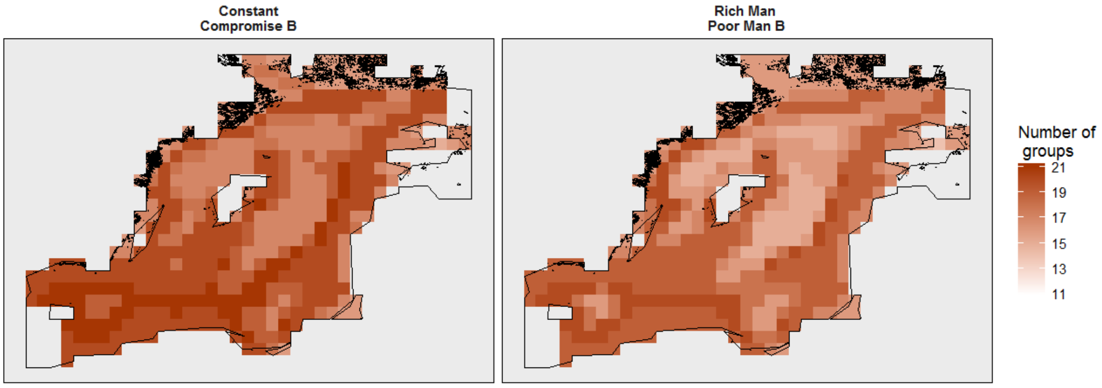

Fig. S8. Species richness in scenarios driven by GCM B outputs.

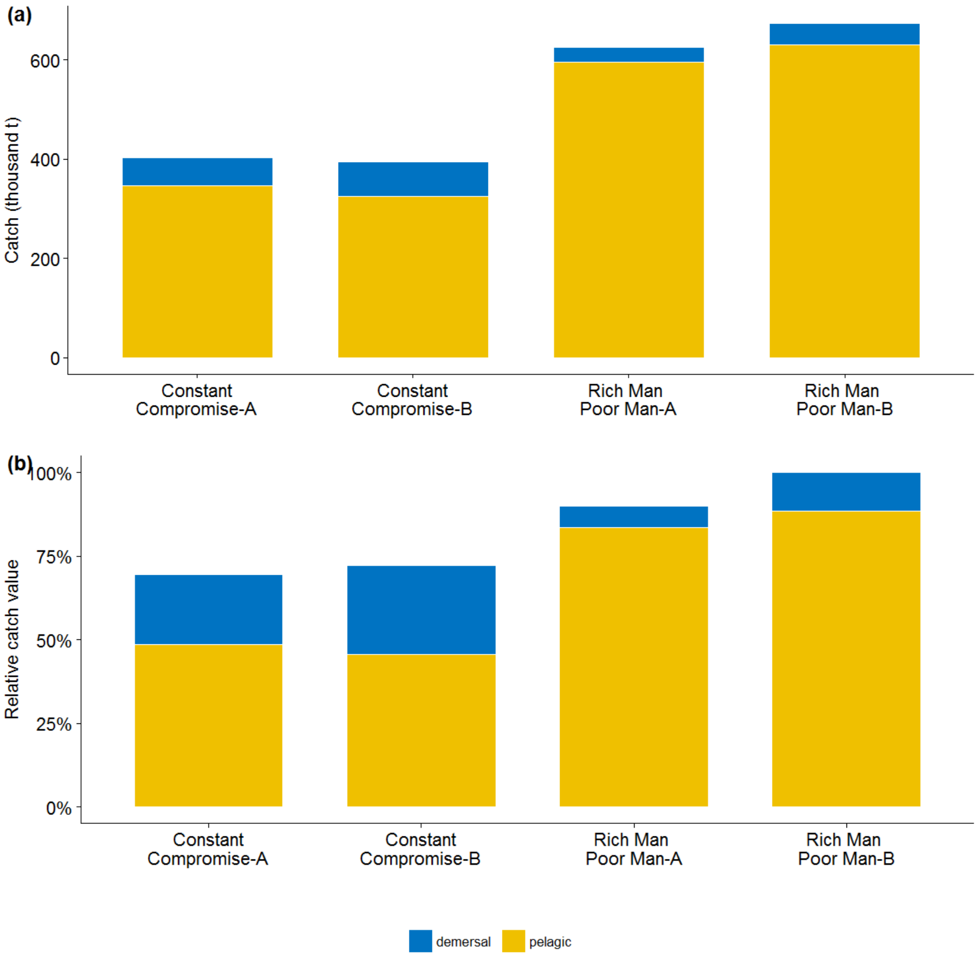

Fig. S9 Catch and relative catch value comparison for models forced by GCM A and GCM B.

S4 Supplementary figures - Sensitivity of fishing effort optimization to fleet economic parameters

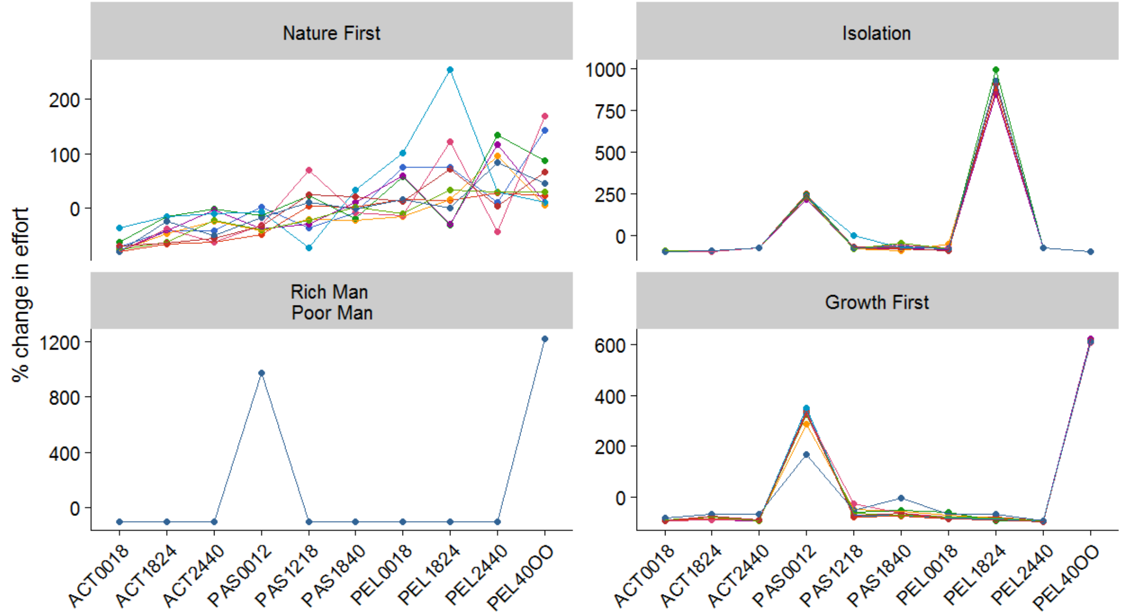

Fig. S10. Efforts estimated by the ‘Fishing policy search’ tool used in scenario simulations, when setting fleet-specific costs. Different colors represent estimates from 10 individual repetitions of the search procedure, each started with random efforts. Differences between repetitions of the search procedure to find optimal fishing efforts for the Rich Man, Poor Man scenario were small and are therefore not visible. In the integrated scenario simulations, the average of the 10 searches shown here were used.

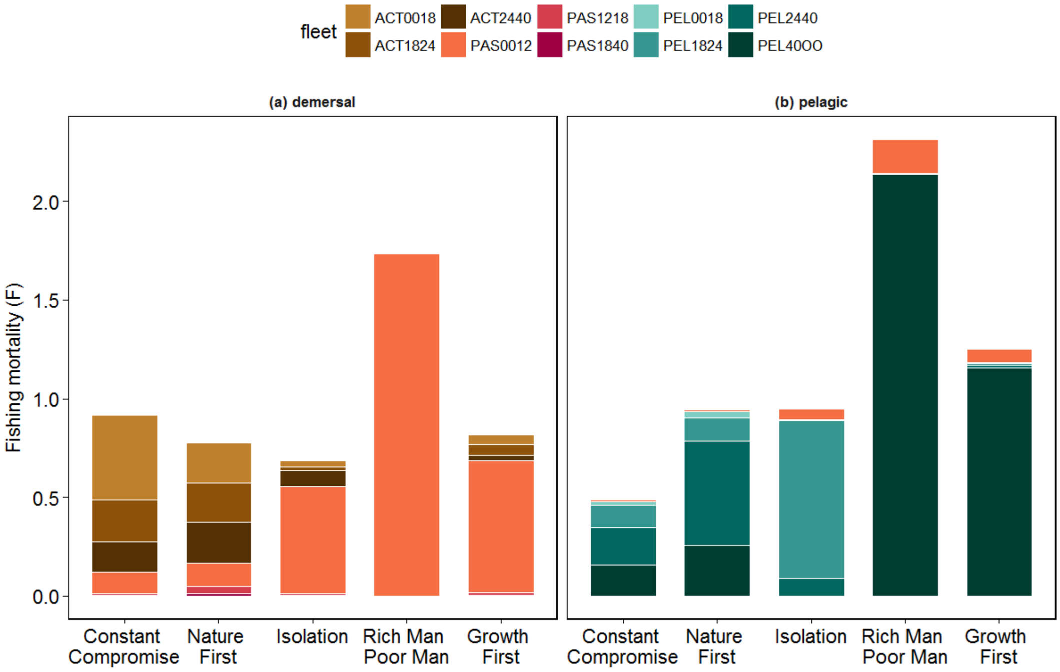

Fig. S11 Fishing mortality rates by fleet resulting from average efforts calculated from the optimal effort search results shown on Fig. S10.

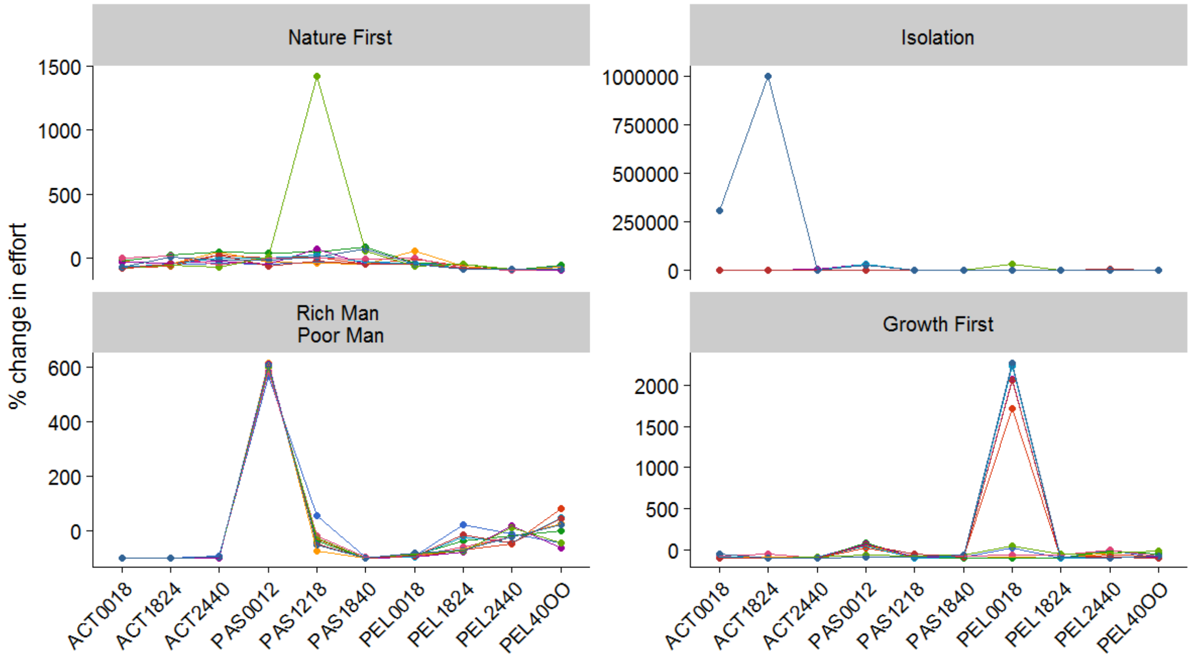

Fig. S12. Efforts estimated by the 'Fishing policy search' tool when setting costs of all fleets equal. Different colors represent estimates from 10 individual repetitions of the search procedure, each started with random efforts. When costs are set equal, only catch composition and relative price of fish caught by the different fleets govern the effort search procedure.
